# Chemical characterization, antioxidant and antidiabetic activities of a novel polyherbal formulation comprising of *Hordeum vulgare*, *Elettaria cardamomum* and *Cicer arietinum* extracts

**DOI:** 10.1016/j.heliyon.2023.e19292

**Published:** 2023-08-19

**Authors:** Rabia Iqbal, Iqbal Azhar, Muhammad Nasir Iqbal, Irfan Hamid, Muhammad Zahoor, Muhammad Furqan Akhtar, Zafar Alam Mahmood, Riaz Ullah, Amal Alotaibi

**Affiliations:** a Department of Pharmacognosy, Faculty of Pharmacy and Pharmaceutical Sciences, University of Karachi, Karachi, Pakistan; b Medical Officer, Tehsil Head Quarter, PD Khan, Jhelum, Pakistan; c Cadson College of Pharmacy, Kharian, University of the Punjab, Lahore, Pakistan; d Department of Biochemistry, University of Malakand, Dir Lower, 18800, Khyber Pakhtunkhwa, Pakistan; e Riphah Institute of Pharmaceutical Sciences, Riphah International University, Lahore Campus, Pakistan; f Department of Pharmacognosy, College of Pharmacy, King Saud University, Riyadh, Saudi Arabia; g Department of Basic Sciences, College of Medicine, Princess Nourah Bint Abdul rahman University, Riyadh, 11671, Saudi Arabia

**Keywords:** Antidiabetic, Antioxidant: polyherbal, HPLC-DAD, Hordeum vulgare, Elettaria cardamomum, Cicer arietinum

## Abstract

Diabetes mellitus (DM) is the most prevalent endocrine disorder. Numerous individual herbs possess anti-diabetic activity. The seeds of *Hordeum vulgare*, *Elettaria cardamomum* and *Cicer arietinum* are traditionally used to manage DM. The ambition of this work was to formulate the poly-herbal granules (PHGs) comprising of these three functional foods and evaluate their *in-vitro* antioxidant and antidiabetic potential. The dried seed extracts of *Hordeum vulgare, Elettaria cardamomum* and *Cicer arietinum* were used in a ratio of 2.5:1:1 to formulate PHGs by wet granulation method. The ratio of extracts was selected on the basis of traditional phytotherapies popularly used by local Hakeems of Pakistan to achieve glycemic control in diabetic patients resistant to traditional allopathic regime of medicine. The flow properties of developed PHGs were evaluated. The UV–Visible spectroscopic, Fourier Transform Infrared (FTIR) and HPLC-DAD of all seed extracts and PHGs were performed. The *in-vitro* antioxidant DPPH, FRAP, total antioxidant capacity (TAC) and Nitric Oxide (NO) scavenging assays were carried out on PHGs. The *in-vitro* antidiabetic activity of PHGs was investigated by alpha-amylase and alpha-glucosidase enzyme inhibition activity. The developed PHGs exhibited excellent flow properties. The UV–Vis spectra of all seed extracts and PHGs demonstrated peak at 278 nm showing the presence of flavonoids and phenols. The FTIR spectra *confirmed the existence of* fl*avonoids, and phenols along with amines in seed extracts as well as PHGs. The HPLC-DAD test revealed the existence of gallic acid, ascorbic acid,* Quercetin-3-(caffeoyldiglucoside)-7-glucoside, *Rosmarinic acid, delphinidin-3,5-diglucosides, Kaempferol-3-feruloylsophoroside-7-glucoside and Phloroglucinol in PHGs. The PHGs exhibited IC*_*50*_ of 51.23, 58.57, 55.41 and 53.13 μg/mL in DPPH assay, FRAP assay, TAC, Nitric oxide scavenging assays respectively. The PHGs also demonstrated IC_50_ of 49.97 and 36.16 μg/mL in alpha-amylase and in alpha-glucosidase inhibition assays respectively in dose dependent manner. The developed PHGs exhibited an excellent flow property. These exhibit significant in-vitro antioxidant and antidiabetic profile by virtue of flavonoid and phenolic acid derivatives.

## 1 Introduction

Diabetes mellitus (DM) is a major global disorder that affects geriatric and immune-compromised population. Multiple factors which contribute to the prevalence of DM and its complications include genetic mutations, unhealthy life style, medical history and side effects of drugs [1]. Genetic mutations and inheritance are the main factors that have increased the prevalence rate of DM. A few genes associated with the occurrence of DM are HLA-DQB1, HLA-DQA1 and HLA-DRB1 genes [2]. These genes instruct the synthetic proteins to take part in the immune system.

According to Center for disease control (CDC), 34.2 million individuals were diagnosed with DM in the USA during 2020. The prevalence rate of DM is proportional to the age. With respect to the ethnicity, a higher rate of DM is observed among non-Caucasians and Asian citizens. Generally, North Americans and European are more prone to DM as compared to Asians. As per report of World Health Organization (WHO) in 2021, the rate of DM is dangerously rising around the globe especially in the middle- and low-income countries. It is estimated to rise up to 693 million by 2045. The population affected by DM in Pakistan is 6.2 million and majority of people are in age range of 20–79 years which indicates that over 11% of the adult population is suffering from DM [3].

An inappropriate secretion or inability of insulin to regulate blood glucose level causes hyperglycemia and other serious symptoms [4]. DM is a complicated metabolic disorder of carbohydrates, fats and proteins characterized by hyperglycemia. DM is categorized into insulin dependent DM (T1DM) and non-insulin dependent DM (T2DM) based upon the degree of pancreatic defects [5].

T1DM or juvenile onset diabetes is an outcome of cell mediated autoimmune destruction of pancreatic β-cells, requiring daily administration of exogenous insulin. T1DM is the consequence of lymphocytic infiltration and obliteration of β-cells. The reduction of β-cell number in turn diminishes the secretion of insulin, while the accessible insulin fails to maintain long-term glycemic control. However, hyperglycemia usually develops after 80–90% destruction of β-cells ensues T1DM. This type of diabetes constitutes to about 5–10% of all cases. The everlasting outcomes of hypoglycemia are tissue and organ dysfunction, which lead to irreversible defects like lack of vision, limb amputation, ischemic stroke and even loss of life. Severe symptoms include diabetic ketoacidosis and hyper-osmolarity that may cause comatose state. T1DM commonly presents symptoms of polyuria, polydipsia, polyphagia, weight gain, fatigue, constipation, cramps, blurred vision and candidiasis [6]. T2DM is more prevalent among adults due to which it is called adult-onset diabetes. T2DM has prevalence of about 90–95% of all DM cases. In this type of diabetes, β-cells of the pancreas are dysfunctional and produce little to no quantity of insulin. It is considered a complex metabolic disorder with varying degrees of insulin resistance. In addition, it alters metabolism of glucose due to the inability of the body in terms of utilizing insulin. The pathophysiology of T2DM involves reduced insulin secretion, exalted production of glucagon, increased gluconeogenesis, neurotransmitter dysfunction and insulin resistance, lipolysis, renal glucose reabsorption, diminished incretin effect, and defective absorption of glucose in muscle, liver-fat signaling axis. Risk factors involved in T2DM include genealogy, overweight, unhealthy diet, lack of exercise, state of ageing, race, hyperglycemia stage of pregnancy, polycystic ovary syndrome, increased blood pressure and imbalance of lipids [7].

There are a variety of management techniques for DM in addition to prescribing insulin. Current available glucose lowering therapies target the key pathways involved in the pathophysiology of type-II diabetes. The considerable number of oral hypoglycemic agents are commonly used. Most synthetic anti-diabetic drugs have severe and long-term side effects such as weight gain, drug resistance, and hypoglycemia as well hepato- and nephrotoxicity. To overcome this challenge, researchers increasingly focus on natural products for future drug development strategies that have more safety, less undesirable effects and are patient friendly.

Plants are feasible source of bioactive agents, like alkaloids, glycosides, flavonoids and polyphenols that mainly exhibit anti-diabetic effect by their anti-inflammatory, anti-glucose and blood lipid regulation properties [8]. Although numerous individual herbs have demonstrated anti-diabetic activity, considerably fewer researches have been conducted on poly-herbal formulations. It is believed that poly-herbal formulations have multiple phytochemicals which have synergistic anti-diabetic effects and can enhance the desired actions [9].

The seeds of *Hordeum vulgare* (Barley) (Family: *Poaceae*), *Elettaria cardamomum* (Cardamon) (Family: *Zingiberaceae*) and *Cicer arietinum* (Gram) (Family: *Fabaceae*) are functional food items and have folkloric usage of anti-inflammation, anti-oxidant and anti-diabetic actions. The local *Hakeems* in Pakistan use these herbs in a respective ratio ***2.5:1:1*** to achieve glycemic control in diabetic patients resistant to traditional allopathic regime of medicine. Reported by Ref. [10]. *Hordeum vulgare* has been previously reported with promising antioxidant and antidiabetic properties [11]. The primary phenolics reported in *H. vulgare* are catechin, ferulic acid and naringin, which are reported to be responsible for the antioxidant-based antidiabetic activity of the *H. vulgare* [11]. Similarly, Al-Yousaf et al., reported the aqueous extract of *Elettaria cardamomum* for its antioxidant and antidiabetic activity. The main compounds found in *E. cardamomum* were phenols, flavonoids, coumarin and anthocyanins [12]. *Cicer arietinum* was found to possess high potential free radical scavenging properties as well anti-hyperglycemic effect in various investigation and abundance of polyphenolic compounds [13]. The high potential of these herbs revealed in previously reported literature and applications in traditional system of remedy, fascinated our interest to explore them for their antidiabetic effect and provide a possible mechanism through *in vitro* antioxidant assays. Therefore, the study was designed to highlight the antioxidant and anti-diabetic properties of popular functional foods and herbs by developing their poly-herbal formulation in addition to chemical characterization. Moreover, to provide a deeper insight, the chemical composition of the herbs was evaluated through UV–vis, FTIR and HPLC-DAD analysis.

## 2 Material and method

### 2.1 Procurement of dried seed extracts

Dried seed extracts of *Hordeum vulgare “Barley”* (Batch #1900613) and *Elettaria cardamomum “Cardamon”* (Batch #20190703) was procured from Changchun people pharmaceutical Group Co.,Ltd, China. Dried seed extract of *Cicer arietinum “Gram”* (Batch #YZD-191021) was obtained from Changsha Natureway Co., Ltd. China. All the extracts were collected in November 2019.

#### 2.1.1 Chemicals and reagents

2,2-diphenyl 1-picrylhydrazyl (DPPH), sodium nitroprusside, sodium potassium tartrate, potassium ferricyanide (sigma-Aldrich, USA), α-glucosidase, alpha-amylase and 3,5-Dinitrosalicylic acid (DNSA) were acquired from Unichem, UK, were used in the current study. Avicel-200, acarbose, ascorbic acid, acetic acid, ammonium molybdate, α-amylase, α-glucosidase, ferric chloride (FeCl_3_), Griess reagent, lactose monohydrate, methylparaben, methanol, 4-nitrophenyl β-d-glucopyranoside (pNPG), propylparaben, potassium bromide, potassium ferricyanide, phosphate buffer, starch, sulfuric acid, sodium nitroprusside, sodium potassium tartrate, sodium phosphate, sodium hydroxide (NaOH), sodium carbonate (Na_2_CO_3_) and tri-chloroacetic acid were used in the study. All these chemicals were purchased from the supplier of Merk and Co. pvt Ltd., and Sigma Aldrich.

### 2.2 Preparation of poly-herbal granules (PHGs)

The formula of PHGs was designed according to Ref. [10]. The dried seed extracts of *Hordeum vulgare* (H.V), *Elettaria cardamomum* (E.C) and *Cicer arietinum* (C.A) were mixed in the ratio of 2.5:1:1. The mixture was triturated and passed through sieve number 40 to obtain homogenized poly-herbal mixture. The trial of PHG preparation was started by adding different ratio of binder and mixing the appropriate quantity of lubricants and preservatives, and finally the procedure was optimized. The final mixture was taken for the preparation of granules by wet granulation using 5% starch paste (binder). The composition of PHGs is mentioned in Table 1. The wet mass was passed through mesh sieve number 22 to obtain uniform size granules. The obtained granules were dried at 45 °C in a tray for 1 h and stored in an air tight labeled container [14].

**Table 1:** Formulation of granules.

Ingredients	Quantity (g)	percentage
*Hordeum vulgare*	12.5	25
*Elettaria cardamomum*	5	10
*Cicer arietinum*	5	10
Avicel-200	16.2	32.4
Lactose monohydrate	10.8	21.6
Methylparaben	0.15	0.3
Propylparaben	0.05	0.1
Starch paste	Quantity Sufficient	–
**Total**	**49.7**	**99.4**

### 2.3 Pre-formulation studies of PHGs

Flow properties of PHGs were determined by standard method [15] and the best trial batch was selected for capsule filling and pre-formulation studies.

#### 2.3.1 Bulk density (δb)

The bulk density was measured by taking weighted quantity (4 g) of the granule blend in a graduated cylinder of 10 mL capacity. The obtained untapped volume of PHGs is bulk volume (V_b_). The bulk density of PHGs was computed by the formula.

δb=Mass/Vb

#### 2.3.2 Tap density (δt)

The bulk mass of the PHGs was mechanically tapped hundredfold from the height of 2.5 cm and after ensuring the settlement of persistent volume, the resultant tapped volume (V_t_) was measured. The tapped density was computed by the formula.

δt=Mass/Vt

#### 2.3.3 Carr's compressibility index

The degree of flow properties of the PHGs was determined by calculating the Carr's compressibility index. It was determined by the given formula.

$$Carr’s\;compressibility\;index=\left(\frac{\delta t-\delta b}{\delta t}\right)\times 100$$

#### 2.3.4 Hausner's index

Hausner's index was determined by dividing the tapped density to bulk density.

#### 2.3.5 Angle of repose (θ)

It was determined by applying the fixed height method. The funnel was elevated to 2 cm height (h). PHGs were poured through a funnel. The radius (r) of the pile was measured and angle of repose was measured by the following formula.

$$Angle\;of\;repose\;\left(\theta \right)=\tan^{-1}\left(\frac{h}{r}\right)$$

### 2.4 UV–Visible analysis of dried seed extracts and PHGs

The UV/Vis analyses of all dried seed extracts and PHGs were performed by UV/Vis spectrophotometer according to the previously described method [16]. The solutions of individual extracts and PHGs were prepared by dissolving 50 mg in 100 mL distilled water separately. The resultant solution was sonicated and filtered through Whatman filter number 1. The samples were scanned in the UV/Vis region at wavelength of 200–800 nm against distilled water as a blank. The characteristic peaks were recorded.

### 2.5 FTIR of dried seed extracts and PHGs

The distinctive functional groups in the individual dried seed extract and PHGs were identified by using Fourier transform infrared (FTIR), Bruker Germany, as per described method [17]. The absorption spectrum of FTIR provided information about the structure of frequently obtained molecules. A slight quantity (10 mg) of the sample was mixed with 100 mg dehydrated potassium bromide (KBr). The blend was efficiently mixed and pressed at 6 bars for 2 min to form thin translucent KBr disc. The sample was then placed in a sample holder of diffuse reflectance accessory tray (Universal ATR). The sample was scanned from 4000 to 450 cm^−1^. The FTIR profiling spectrum of all the samples were collected.

### 2.6 HPLC-DAD analysis of extracts and PHGs

#### 2.6.1 Preparation of sample

For quantitative analysis of phytochemical contents in the PHGs, and dried seed extracts of H.V, E.C and *C.A, HPLC-DAD analysis was performed according to method described previously* [18]*.* The sample was made by combining 20 mL of methanol and water (1:1) with 1 g of each sample. The sample was kept in a water bath for 1 h to heat it at 70 °C. This sample was allowed to cool and filtered with a syringe filter to obtain 2 mL aliquot following centrifugation at 4000 rpm for 10 min.

#### 2.6.2 HPLC-DAD analysis

HPLC equipped with auto-sampler, degasser and diode array detector (DAD) was used to identify numerous compounds. The compounds were separated by Agilent Zorbax Eclipse XDB-C18 column. The gradient system contains solvent A having methanol, acetic acid and deionized water in a respective ratio of 100:20:180 and solvent B in a respective ratio of 900:20:80. After 25 min, elution occurred. For the analysis of phenolic compounds, the DAD was set at 320 nm to record the spectra.

### 2.7 In-vitro antioxidant studies

#### 2.7.1 DPPH antioxidant assay

It was carried out to demonstrate the free radical scavenging activity of PHGs and different extracts by adopting the previous method [19]. The freshly prepared 2 mL methanolic solution of DPPH (0.1 mM) was mixed with 2 mL of PHGs at different concentrations. The resulting solution was shaken well and kept in dark at 25 °C for 0.5 h. Then, this mixture was centrifuged at 1500 rpm for 10 min, after which, the absorbance of (supernatant) was recorded at 517 nm by UV–Vis spectrophotometer. Methanol served as blank and ascorbic acid was utilized as standard antioxidant. The procedure was performed in triplicate. The scavenging capability of PHGs was measured and IC_50_ was calculated.

#### 2.7.2 Ferric (Fe3+) reducing power activity (FRAP) assay

The FRAP assay, a high throughout method, was used to confirm the antioxidant capability of PHGs based on reduction of the colorless oxidized ferricyanide by electron donation [20]. An aliquot of 100 μL of PHGs solution was mixed with 200 μL of phosphate buffer (0.2 mol/L, pH 6.6) and 250 μL potassium ferricyanide (1% w/v in H_2_O). The resultant mixture was shaken vigorously, and incubated at 50 °C for 20 min. It was then centrifuged at 3000 rpm at 25 °C for 10 min after addition of 200 μL of tri-chloroacetic acid (10% w/v in H_2_O). The obtained supernatant (150 μL) was mixed with 50 μL of FeCl_3_ (0.1% w/v in H_2_O). The absorbance was recorded at 630 nm by UV–Vis spectrophotometer. Methanol was used as a blank and ascorbic acid was utilized as standard agent. The procedure was repeated three times. The IC_50_ of PHGs was calculated and compared with that of ascorbic acid.

#### 2.7.3 Total antioxidant capacity (TAC)

The TAC of PHGs was assessed by phosphomolybdate assay according to a previous method in which antioxidants reduce molybdenum to a green colored reduced form [21]. A 100 μL of freshly prepared PHGs solution or standard was mixed with 900 μL of phosphomolybdate reagent. The reaction mixture was incubated at 95 °C for 90 min. The absorbance of PHGs and ascorbic acid solutions was measured at 630 nm by UV–Vis spectrophotometer after cooling at 25 °C. Methanol was used as blank. The experiment performed in triplicate. The total antioxidant activity was calculated and reported as μg of ascorbic acid equivalents (AAE) per mL.

#### 2.7.4 Nitric oxide free radical scavenging assay

This assay is used to determine the nitric oxide scavenging activity of samples such as flavonoids. This assay was performed by the method described previously with minor changes [22]. The stock solution of PHGs or standard (ascorbic acid) was freshly prepared in methanol. A range of concentrations of PHGs or standard (20–100 μg/mL) was mixed with 2 mL of 10 mM sodium nitroprusside which was prepared in 0.5 mM phosphate buffer saline (pH 7.4). The obtained solutions were incubated for 3 h at 25 °C. After incubation, 0.5 mL solution of PHGs or standard was mixed with equal volume of Griess reagent. The absorbance of the PHGs or ascorbic acid solution was recorded at 546 nm by UV–Vis spectrophotometer. The percentage inhibition and IC_50_ of nitric oxide by PHGs and standard were calculated.

### 2.8 In-vitro anti-diabetic activity

#### 2.8.1 Alpha amylase inhibition

This assay was carried out on various concentrations of PHGs to determine the *in-vitro* antidiabetic potential [23]. A well-known standard drug acarbose was used as inhibitor of α-amylase enzyme. To produce DNSA reagent, 2.36 g of DNSA was mixed in 80 mL of 0.5 mol/L of sodium hydroxide with subsequent addition of 30 g sodium potassium tartrate. Briefly, starch solution of 1% *w/v* was prepared followed by addition of 0.02 M phosphate buffer to set the pH of solution at 6.9. To continue the procedure, an equivalent quantity of α-amylase (1% *w/v*) and PHGs solution were mixed and kept for 0.5 h at room temperature. Afterwards, 1 mL starch solution was added up and the resultant mixture was incubated for 10 min at 25 °C. Then, 1 mL DNSA reagent was put in this solution after heating for half an hour at 90 °C on water bath and the final volume was made up to 10 mL with water. The absorbance of individual sample was recorded at 540 nm. The percentage inhibition of alpha Amylase was determined [24].

#### 2.8.2 Alpha glucosidase assay

This assay is based on inhibition of alpha-glucosidase activity and was performed with some alterations in the previous protocol [25]. Briefly, 20 μL of different dilutions of PHGs were mixed with 10 μL of α-glucosidase 1U/mL and 125 μL of 0.1 M phosphate buffer (pH 6.8). This mixture was incubated for 20 min at 37 °C. To start the reaction, a solution of 20 μL of 1 M 4-Nitrophenyl β-d- glucopyranoside (pNPG) as a substrate was added, and incubated for 30 min. To stop the reaction, 50 μL of 0.1 N Na_2_CO_3_ was added. The spectrophotometric absorbance was measured at 405 nm. Acarbose was utilized as a standard. The experiment was repeated in triplicate. The percentage inhibition of α-glucosidase was recorded.

### 2.9 Statistical analysis

All the assays and experiments were carried out 5 times and the results were demonstrated as mean average ± standard deviation (Std.) n = 5. One-way *ANOVA* followed by Tukey's multiple comparison test, was applied for statistical analysis. The data was analyzed by using Graphpad prism 7.0 software to calculate IC-50.

## 3 Results

### 3.1 Flow properties of PHGs

The physicochemical characters of PHGs were confirmed by bulk density, tapped density, Carr's compressibility index, Hausner's ratio and angle of repose (Table 2). The values of bulk density and tapped density were used to determine the Carr's compressibility index and Hausner's ratio. The Carr's compressibility index of PHGs was 3.170 ± 0.01 that was below the described limit of USP standards (≤10). Hausner's ratio of PHGs was calculated to be 1.033 ± 0.01 that is also within limit of USP standards (1.00–1.11). The value of angle of repose for PHGs was 28.07 ± 0.01° that is within range of 25–30 as per USP standards. These parameters indicated that the PHGs exhibited excellent flow properties with respect to the defined standard ranges of USP.

**Table 2:** Flow properties of polyherbal granules.

Flow Properties	PHGs values	USP Standards
*Bulk density*	0.633 ± 0.14 g/mL	–
*Tapped density*	0.654 ± 0.21 g/mL	–
*Carr's Compressibility index*	3.170 ± 0.01%	≤10
*Hausner's index*	1.033 ± 0.01	1.00–1.11
*Angle of repose*	28.07 ± 0.01^◦^	25–30^◦^

All the data presented in Mean ± SD (n = 5).

### 3.2 UV–vis analysis of dried seed extracts and PHGs

The qualitative UV–Vis spectra of dried seed extracts of H.V, C.A, E.C and PHGs showed the peak at wavelength 278 nm with the absorption of 0.535, 0.545, 0.356 and 0.430 respectively (Table 3). Previous studies showed that the absorption bands at range of 234–676 nm were characteristic of the alkaloids, flavonoids and phenolic compounds [16]. In this case, peaks in all the samples appeared at 278 nm which suggested the presence of these secondary metabolites.

**Table 3:** UV–Vis spectrum of the dried seed extracts and PHGs indicating the presence of secondary metabolites.

Sample	Absorbance	Wavelength (nm)	Reference wavelength range (nm)	Phytochemical compounds
*Hordeum vulgare*	0.535	278	234–676	Flavonoids, alkaloids and phenolic compounds
*Cicer arietinum*	0.545	278		
*Elettaria cardamomum*	0.356	278		
Poly herbal granules	0.430	278		

### 3.3 Interpretation of FTIR spectra of dried seed extracts and PHGs

FTIR spectra were used to detect the functional groups of the active constituents in the samples on the basis of peak values in the infrared region. The single bond, double bond and fingerprint regions in each sample indicated specific functional groups (Table 4). The peaks of single bond region in the IR spectra between *3200–*3400 cm^−1^ *and 2800–*3000 cm^−1^ *demonstrated OH and CH groups respectively. The peaks of double bond region in the IR spectra between 1600–*1800 cm^−1^ *indicated the presence of carbonyl compounds of amides and esters in respective samples. The fingerprint area between 1310-*1410 cm^−1^ *pointed out the presence of phenol or tertiary alcohol (OH bend), sulfonate compounds and aromatic nitro compounds. Moreover, the peaks between 1000-*1200 cm^−1^ *represented the primary amines (C–N stretch) and aliphatic fluoro-compounds (C–F stretch). Furthermore, the peaks located in the area of 500-*1000 cm^−1^ *provided information about the existence of thiols and thio-substituted compounds of disulfides (C–S stretch, S–S stretch), aliphatic bromo and chloro compounds (C–Br stretch and C–Cl stretch), aromatic phosphates compounds (P–O–C stretch) and alcohol (OH out of plane bend)* [26]*.* The FTIR spectrum of the dried seed extracts of H.V, EC, C.A and PHGs are shown in Table 4.

**Table 4:** Mid IR spectrum regions of FTIR and its quantified frequencies in the dried seed extracts and Polyherbal granules.

Different regions	IR Ranges (cm^−1^)	Peak Origin of samples				Functional Groups and Compounds
		*Hordeum vulgare*	*Elettaria cardamomum*	*Cicer arietinum*	Polyherbal granules	
Single bond region (2500-4000 cm^−1^)	3200–3400	3315	3309.5	3323.5	3322.5	O–H group in alcohols and phenols
	2800–3000	2919.5	2913.5	2927	2899.5	C–H stretching of alkane or alkyl groups
Double bond region (1500-2000 cm^−1^)	1600–1800	1635.5	1642	1635	1640.5	Carbonyl compounds of amides and esters
Fingerprint region (600-1500 cm^−1^)	1310–1410	1346	1352.5	1340.5	1370	Phenol or tertiary alcohol, OH bends, sulfonate compounds, aromatic nitro compounds.
	1000–1200	1011	1014	1004.5	1110	Primary amines (C–N stretch) and Aliphatic fluoro compound (C–F stretch).
	570–705 590–720 850–995	609	570	571	988.5	Thiols and thio-substituted compounds of disulfides (C–S stretch). Alcohol, (OH out-of-plane bend). Phosphorus oxy compounds; Aromatic phosphates (P–*O*–C stretch).
	700–800				774	Aliphatic chloro-compounds, (C–Cl stretch).
	600–700				602.5	Thiols and Disulfides (S–S stretch), aliphatic and bromo compounds (C–Br stretch).

### 3.4 HPLC-DAD analysis

The estimation of phenolic compounds presents in E.A, H.V, C.A and PHGs, was performed by HPLC-DAD analysis*. The compounds were detected by HPLC-DAD through comparison with standard compounds and those reported in literature based on coincidences in retention time.*

Table 5, Table 6, Table 7, Table 8 represent the typical chromatogram of all three extracts and PHGs. It was found that Quercetin-3,7-di-*O*-glucoside and Kaempferol 3-(caffeoyldiglucoside)-7-rhamnosy were found in the highest amount in H.V, while Syringic acid and pyrogallol were detected in the highest amount in E.C. Moreover, Vanillic acid-hexoside and Kaempferol-3-(*p*-coumaroyldiglucoside)-7-glucoside were detected in the highest amount in C.A. HPLC-DAD analysis demonstrated that the PHGs were abundant in Delphinidin-3,5-diglucoside, Ascorbic acid and Delphinidin-3,5-diglucoside as shown in Table 8.

**Table 5:** Phenolic profile of dried seed extract of *Hordeum vulgare* determined by HPLC-DAD.

Peak	Rt (Min)	Identity	Amount (μg/g)
1	2.665	Malic acid	39.73
3	22.284	Rutin	118.06
4	22.934	Quercetin-3,7-di-*O*-glucoside	3846.81
5	24.447	Quercetin-7-*O*-sophoroside	19.58
7	25.470	Kaempferol 3-(caffeoyldiglucoside)-7-rhamnosy	2266.49
9	27.398	4-Caffeoyl-5-coumaroylquinic acid	446.62
10	27.785	Kaempferol-3-(*p*-coumaroyldiglucoside)-7-glucoside	742.58
11	29.817	Caffeic acid	182.50
13	31.034	Mandelic acid	47.08
14	31.436	Quercetin-3-(caffeoyldiglucoside)-7-glucoside	91.07
15	32.027	Delphinidin-3,5-diglucoside	24.68
19	35.127	Galloyl-bis-HHDP-hex (casuarinin)	152.56
20	35.610	Phloroglucinol	24.57
21	36.118	Hydroxy benzoic acid	64.09
22	38.682	5-*O*-Caffeoylquinic acid	47.90
23	44.137	Dihydro kaempferol-hexosides	132.68
*Totals*			**8247**

**Table 6:** Phenolic profile of dried seed extract of *Elettaria cardamomum* determined by HPLC-DAD.

Peak	Rt (Min)	Identity	Amount (μg/g)
1	2.697	Ascorbic acid	84.03
2	16.290	Ellagic acid	32.80
3	17.068	Kaempferol-3-(*p*-coumaroyldiglucoside)-7-glucoside	57.25
4	22.596	Rutin	71.64
5	23.330	Syringic acid	2516.60
6	25.106	Kaempferol 3-(caffeoyldiglucoside)-7-rhamnosyl	79.01
8	27.578	4-Caffeoyl-5-coumaroylquinic acid	279.70
9	27.951	Pyrogallol	495.30
10	29.943	Caffeic acid	77.47
11	31.104	Mandelic acid	30.50
12	31.509	Quercetin-3-(caffeoyldiglucoside)-7-glucoside	67.91
13	32.095	Delphinidin-3,5-diglucoside	14.09
18	35.191	Galloyl-bis-HHDP-hex (casuarinin)	149.23
19	35.684	Phloroglucinol (35.4)	24.73
20	36.205	Hydroxy benzoic acid	60.67
21	38.816	5-*O*-Caffeoylquinic acid	46.53
22	44.317	Dihydro kaempferol-hexosides	117.48
*Totals*			**4204.94**

**Table 7:** Phenolic profile of dried seed extract of *Cicer arietinum* determined by HPLC-DAD.

Peak	Rt (Min)	Identity	Amount (μg/g)
1	2.648	Malic acid	235.70
2	3.468	Galloyl-HHDP-glucoside (lagerstannin C)	33.33
3	5.365	bis-HHDP-hexoside (pedunculagin I)	18.13
4	6.181	Gallic acid	13.25
5	6.574	Digalloyl-hexoside	20.15
9	13.692	Protocatechuic acid	30.40
13	17.231	Caffeic acid derivative	65.80
15	18.752	HHDP-gallagyl-hexoside (punicalagin)	28.07
19	22.304	Rutin	287.70
20	23.045	Vanillic acid-hexoside	4244.42
22	24.990	Kaempferol 3-(caffeoyldiglucoside)-7-rhamnosy	87.61
25	27.467	4-Caffeoyl-5-coumaroylquinic acid	442.99
26	27.852	Kaempferol-3-(*p*-coumaroyldiglucoside)-7-glucoside	740.09
27	29.489	Caffeic acid	32.13
29	30.078	Caffeic acid hexoside	121.84
30	30.778	Ellagic acid derivative	26.35
31	31.064	Mandelic acid	46.39
32	31.467	Quercetin-3-(caffeoyldiglucoside)-7-glucoside	90.81
34	32.388	Delphinidin-3,5-diglucoside	26.96
35	33.174	Caffeic acid hexoside derivative	9.33
37	35.153	Digalloyl-HHDP-gluc (punigluconin)	151.96
38	35.641	Phloroglucinol	24.45
39	36.154	Hydroxy benzoic acid	64.20
40	38.734	Ellagic acid derivatives	46.83
41	44.200	Brevifolin carboxylic acid	130.98
*Totals*			7019.87

**Table 8:** Phenolic profile of polyherbal granules determined by HPLC-DAD.

Peak	Rt (Min)	Identity	Amount (μg/g)
1	2.292	Gallic acid	84.01
2	2.795	Ascorbic acid	297.20
3	3.507	Gallic acid derivative	3273.23
4	32.875	Quercetin-3-(caffeoyldiglucoside)-7-glucoside	42.58
5	34.250	Rosmarinic acid	104.02
6	34.935	Delphinidin-3,5-diglucoside	150.75
7	35.401	Kaempferol-3-feruloylsophoroside-7-glucoside	24.52
8	35.882	Phloroglucinol	118.66
*Totals*			4094.97

### 3.5 In-vitro antioxidant activities

#### 3.5.1 DPPH antioxidant assay

**Fig. 1:**
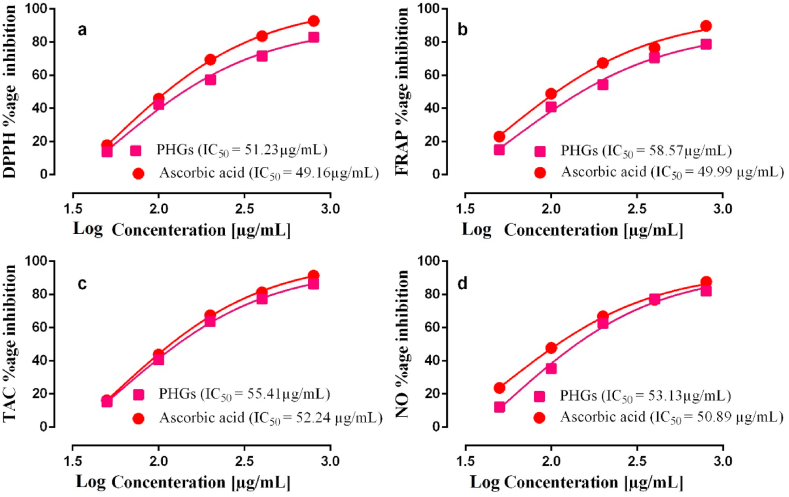
The in-vitro antioxidant activity of polyherbal granules (PHGs) (a) 2,2-diphenyl 1-picrylhydrazyl (DPPH) scavenging activity, (b) Ferric reducing power activity (FRAP) inhibition activity (c) Total antioxidant capacity (TAC) (d) Nitric oxide (NO) scavenging activity assay.

The percentage inhibition of free radicals by PHGs was found to be 83.09 ± 0.10 at 3200 μg/mL concentration while displaying IC_50_ value of 51.23 μg/mL. The free radical scavenging activity of PHGs was statistically significant (***p < 0.001) compared to the standard ascorbic acid having percentage inhibition of 92.84 ± 0.06 at 3200 μg/mL concentration with IC_50_ value of 49.16 μg/mL (Fig. 1a).

#### 3.5.2 Ferric (Fe^+3^) reducing power activity

The significant (***p < 0.001) reducing power percentage values of PHGs and standard ascorbic acid at concentration of 3200 μg/mL were found to be 78.85 ± 0.06 and 89.86 ± 0.06% with IC_50_ values of 58.57 μg/mL and 49.99 μg/mL respectively. The FRAP results of PHGs are shown in Fig. 1b.

#### 3.5.3 TAC

The total antioxidant capacity of PHGs and ascorbic acid was determined by the phosphomolybdate assay method. The percentage inhibition of free radicals by PHGs and ascorbic acid at concentration of 3200 μg/mL were 86.40 ± 0.10 and 91.44 ± 0.15% respectively with IC_50_ values of 55.41 μg/mL and 52.24 μg/mL. The effect of PHGs was found statistically significant (***p < 0.001) compared to the standard. The TAC of PHGs is displayed in Fig. 1c.

#### 3.5.4 Nitric oxide scavenging activity

Nitric oxide scavenging activity of PHGs and ascorbic acid was assessed which showed concentration dependent increase (***p < 0.001) in scavenging activity. The percentage inhibition of free radicals by PHGs and ascorbic acid at concentration of 3200 μg/mL were 82.10 ± 0.10 and 87.69 ± 0.05% μg/mL respectively, while both displayed the respective IC_50_ values of 53.13 and 50.89 μg/mL (Fig. 1d).

### 3.6 In-vitro antidiabetic activities

#### 3.6.1 Alpha amylase inhibition

**Fig. 2:**
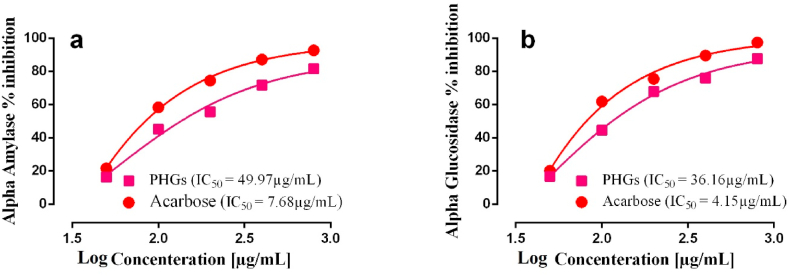
In-vitro antidiabetic activities of poly-herbal granules (PHGs) (a) Alpha amylase inhibition activity (b) Alpha glucosidase inhibition activity of PHGs.

The alpha-amylase inhibitory potential of PHGs was assessed in comparison to standard α-amylase inhibitor (acarbose). The PHGs inhibited (***p < 0.001) the alpha-amylase enzyme in a dose dependent manner like acarbose. Nevertheless, the percentage inhibition of PHGs (81.66 ± 0.06%) was lower than the acarbose (92.65 ± 0.12%) at 3200 μg/mL concentration. The IC_50_ of PHGs was 49.97 μg/mL in comparison to the IC_50_ of Acarbose 7.68 μg/mL (Fig. 2a).

#### 3.6.2 Alpha glucosidase inhibition

The alpha-glucosidase inhibitory potential of PHGs was also assessed in comparison to standard α-glucosidase inhibitor (Acarbose). The PHGs inhibited the alpha-glucosidase enzyme in a dose dependent manner like Acarbose. However, the percentage inhibition of PHGs (87.53 ± 0.15%) was lower than the Acarbose (97.31 ± 0.20%) at 3200 μg/mL. The IC_50_ of PHGs was 36.16 μg/mL against that of acarbose (4.107 μg/mL) (Fig. 2b). The effect of PHGs was observed to be statistically significant (***p < 0.001) compared to the standard.

## 4 Discussion

The poly-herbal formulations contain multiple medicinal plants possessing variety of phytochemical constituents that act synergistically to exhibit pharmacological action. This study developed the PHGs comprising of dried seed extracts of H.V, E.C and C.A. These functional herbs are used in daily life and therefore their developed poly-herbal granules were investigated for their phytochemical constituents, antioxidant properties and antidiabetic potential.

Physicochemical properties of the PHGs prepared by wet granulation method were evaluated which showed that the flow properties were acceptable as per USP standards. *All these parameters indicated the excellent flow properties of prepared PHGs with respect to standard ranges.*

*The UV–Vis spectrum is used to identify the compounds having* π-bond, lone pairs of electrons, sigma-bonds, aromatic rings and chromophores from 200 to 800 nm of UV–Vis range. The peak at 278 nm was observed in all extracts and PHGs which is correlated to the transition of electrons from n–π*. This electron transition in extracts and PHGs demonstrates the presence of hydroxyl, carbonyl, aliphatic and aromatic compounds. The sample with higher antioxidant capacity showed high absorbance at 280 and 358 nm [27]. UV–Vis analysis of plant extracts in reported literature forecasts that absorption bands in range of 234–676 nm are characteristic for alkaloids, flavonoids and phenolic compounds [16]. Thus, UV–Vis analysis of seed extracts and PHGs confirmed the coexistence of various metabolites like alkaloids, flavonoids and phenolics in PHGs.

Moreover, FTIR spectra were analyzed to find out the chemical structure of compounds in dried seed extracts and PHGs. The reported literature on FTIR spectra has confirmed that the peaks of single bond region in the IR spectra between *3200–*3400 cm^−1^ *and 2800–*3000 cm^−1^ *demonstrated the presence of alcohols, phenols and methylene as a result of OH groups and CH symmetric as well as asymmetric vibrations (C–H stretch)* [14]*. The peaks of double bond region in the IR spectra between 1600–*1800 cm^−1^ *indicated the presence of carbonyl compounds of amides and ester in respective samples. The fingerprint area between 1310-* 1410 cm^−1^ *points out the presence of phenol or tertiary alcohol (OH bend), sulfonate compounds and aromatic nitro compounds whereas, the peaks between 1000-*1200 cm^−1^ *represented primary amines (C–N stretch) and aliphatic fluoro-compounds (C–F stretch). Moreover, the peaks located in the area of 500-* 1000 cm^−1^ *provided information about the existence of thiols compounds of disulfides (C–S stretch, S–S stretch), aliphatic bromo and chloro-compounds (C–Br stretch and C–Cl stretch), P–O–C stretch) and alcoholic group* [26]*. The FTIR spectra of dried seed extracts and PHGs showed their peaks in single bond region which confirmed the presence of OH groups of alcohol and phenol as well as another peak in the same region indicated the presence of C–H stretching of alkane and alkyl groups. Similarly, the peaks of all samples in double bond region were evident of the presence of carbonyl compounds of amides and esters. The FTIR spectral peaks of all seed extracts and PHGs were also located in fingerprint region that indicated OH bend of phenol, tertiary alcohols and aromatic compounds, C–N stretch of amines, C–F strech, C–S stretch and S–S stretch of disulfides, C–Br stretch, C–Cl stretch of aliphatic bromo and chloro compounds, P–O–P stretch of aromatic phosphate compounds and OH out of plane bend of alcohols. These specific peaks of various functional groups confirmed the presence of flavonoids, phenols and amines in seed extracts and PHGs.*

*The bioactive compounds were further identified by HPLC-DAD analysis and those reported in literature based on coincidence of retention time* [28]*. The HPLC-DAD analysis of prepared PHGs confirmed the presence of gallic acid, ascorbic acid,* Quercetin-3-(caffeoyldiglucoside)-7-glucoside*, Rosmarinic acid, delphinidin-3,5-diglucosides, Kaempferol-3-feruloylsophoroside-7-glucoside and Phloroglucinol. Gallic acid is a naturally occurring secondary metabolite that belongs to phenolic acids and has antioxidant property* [29]*. Ascorbic acid is common antioxidant* [30]*.* Quercetin-3-(caffeoyldiglucoside)-7-glucoside is a poly-phenolic flavonoid derivative with potent antioxidant activity [31]. The reported rosmarinic acid in PHGs is a natural polyphenol that is an ester of caffeic acid and 3,4 dihydroxyphenyl lactic acid [32]. Delphinidin-3,5-diglucoside was also detected in PHGs which is an anthocyanins flavonoid compound [33]. The Kaempferol-3-feruloylsophoroside-7-glucoside is another main flavonoid compound detected in the PHGs [34]. Phloroglucinol, detected in PHGs, belongs to an essential class of natural products having 1,3,5-trihydroxy benzene with strong antioxidant activity [35]. *The dried seed extracts of H.V, E.C and C.A* were also rich in similarly related bioactive compounds *as confirmed by HPLC-DAD analysis. These phytochemicals detected in dried seed extracts and PHGs* are good source of potent antioxidants like amides, flavonoids and phenols that are useful in the treatment of various diseases, including DM [36]. The reported literature and previous research indicate that antioxidant phytochemicals are useful candidates for the management of DM. So, for the validation of the antioxidant capacity of PHGs, *in-vitro* antioxidant assays like DPPH, Fe^+3^ reducing power, total antioxidant and nitric oxide free radical scavenging activities were performed in comparison to ascorbic acid [37].

The DPPH is frequently utilized as a testing agent to estimate the free radical scavenging action. In this assay, PHGs exhibited inhibition of free radicals. The antioxidant scavenging of the radicals and the cleavage of the hydrogen atom by PHGs is because of greater quantity of phenolic compounds [38]. The reducing power of PHGs was estimated by Fe^+3^ reducing power activity. The FRAP assay quantify the reducing capacity on the basis of ferric ion reduction. The antioxidant acts by radical quenching (H transfer) to reduce the ferric 2,4,6-tripyridyl-*s*-triazine complex [Fe^+3^(TPTZ)_2_]^+3^ to intensely blue colored ferrous complex [Fe^+2^(TPTZ)_2_]^+2^ in acidic medium [39]. The reducing power of PHGs may therefore serve as a significant indicator of its potential antioxidant activity. The TAC of PHGs was demonstrated by phosphomolybdenum method which is based on the reduction of Mo^+6^ to Mo^+5^ by the PHGs and subsequent formation of a green phosphate Mo^+5^ complex at acidic pH [40]. These results of TAC clearly revealed that PHGs have antioxidant potential similar to ascorbic acid. The NO free radical scavenging technique was used to determine the ability of PHGs to prevent the NO induced nitration of 4,5-diaminofuorescein. The results are expressed as percentage inhibition of 4,5-diaminofuorescein oxidation as a function of concentration of NO scavenging [37]. The compounds which contain thiols group displayed a considerable NO scavenging capacity. The FTIR spectra predicted that PHGs contained thiols or thiols substituted compounds in fingerprint region at 602.5 nm which is attributed to NO scavenging capacity of PHGs.

The *in-vitro* antidiabetic potential of PHGs was assessed by alpha amylase and alpha glucosidase inhibition in comparison of standard acarbose. The alpha amylase enzyme is an essential component in the digestion process found primarily in saliva and pancreatic juice and participates in the breakdown of polysaccharides. Acarbose and other α-amylase inhibitors reduce the level of glucose by delaying the digestion of carbohydrates. Targeting this enzyme is one of the possible mechanisms to prevent high postprandial blood glucose [41]. The *in-vitro* inhibition of alpha-amylase by PHGs clearly indicated that PHG might exhibit strong anti-diabetic effects. The alpha glucosidase enzyme is another essential enzyme of the digestion, present in the mucosal brush border of the small intestine. It plays a pivotal role in processing and degradation of complex carbohydrates into smaller, simpler and absorbable ones. The inhibition of alpha-glucosidase is an effective way to delay glucose absorption which ultimately prevents high postprandial blood glucose level and suppresses DM progression [41]. PHGs demonstrated moderate inhibition of alpha-glucosidase which is one of the proposed potential modes of action exhibited by PHGs in the treatment of DM [42].

## 5 Conclusion

The result of this study proved that PHGs comprising of *Hordeum vulgare, Elettaria cardamomum and Cicer arietinum* contained potential polyphenolic phytochemicals like flavonoids and phenolic acids. The *in-vitro* antioxidant activities of PHGs were confirmed due to scavenging of free radicals. Moreover, the PHGs exhibited their antidiabetic potential through inhibition of carbohydrate hydrolyzing enzymes i.e., alpha amylase and alpha glucosidase. It is suggested that *in-vivo* studies must be carried out to validate the antidiabetic potential of these PHGs.

## Author contribution statement

Rabia Iqbal: Irfan Hamid: Conceived and designed the experiments; Performed the experiments; Analyzed and interpreted the data; Wrote the paper.

Iqbal Azhar: Conceived and designed the experiments.

Muhammad Nasir Iqbal: Muhammad Zahoor: Performed the experiments.

Muhammad Furqan Akhtar: Analyzed and interpreted the data; Wrote the paper.

Zafar Alam Mahmood: Riaz Ullah: Amal Alotaibi: Contributed reagents, materials, analysis tools or data.

## Data availability statement

Data will be made available on request.

## Funding statement

Princess Nourah bint Abdulrahman University Researchers Supporting Project (number PNURSP2023R33), 10.13039/501100004242Princess Nourah bint Abdulrahman University, Riyadh, Saudi Arabia.

## Declaration of competing interest

The authors declare the following financial interests/personal relationships which may be considered as potential competing interests:Muhammad Furqan Akhtar serves as Advisory board member of Heliyon Journal.
